# Sustainable Biocatalytic System for the Enzymatic Epoxidation of Waste Cooking Oil

**DOI:** 10.3390/ma17184518

**Published:** 2024-09-14

**Authors:** Iunia Podolean, Madalina Tudorache

**Affiliations:** Department of Organic Chemistry, Biochemistry and Catalysis, Faculty of Chemistry, University of Bucharest, 4-12 Regina Elisabeta Av., 030018 Bucharest, Romania; iunia.podolean@chimie.unibuc.ro

**Keywords:** waste cooking oil (WCO), enzymatic epoxidation, immobilized lipase, system reusability, biocatalyst recyclability

## Abstract

The present study is integrated in a global effort to capitalize waste cooking oil (WCO) into versatile compounds by introducing an oxirane ring into the unsaturated carbon chain of fatty acid residues (the epoxidation of double bound). Therefore, an enzymatic method was set up for the epoxidation of artificially adulterated WCO (SFw) and WCO under real conditions (SFr) derived from sunflower biomass. Commercial lipase (Novozyme, NZ) was used as a biocatalyst for generating the peracid requested by the epoxidation pathway. Optimum experimental conditions (e.g., 1.5 wt% NZ, 1:1:0.5 = H_2_O_2_/double bonds/peracid precursor (molar ratio) and 12 h reaction time) allowed for the conversion of 90% of the SFw substrate into products with an oxirane ring. Octanoic acid was selected as the best peracid precursor. The versatility of the developed system was tested for olive, milk thistle, hemp and linseed oils as both fresh and WCO samples. The characterization of the oil samples before and after the enzymatic epoxidation allowed for the evaluation of the system performance. SFw/SFr exhibited a better susceptibility to enzymatic epoxidation. In addition, the reusability of the biocatalytic system was investigated. Furthermore, different strategies, such as biocatalyst coating and the addition of organic solvents/buffers were applied, limiting enzyme leaching, for the better recovery of the biocatalyst activity.

## 1. Introduction

Nowadays, the valorization of different types of wastes into value-added materials is one of the major concerns, both in the industry and in the research field, due to the emerging depletion of resources on the one hand, and the need to address the continuously damaged environment on the other hand. In this regard, waste cooking oil (WCO) is one of the most important categories of waste that can be successfully capitalized using different methodologies [1,2,3]. The WCO amount generated worldwide is difficult to estimate due to the lack of systematic reporting, especially from the household sector. However, it is certain that the amount of this type of waste will continue to increase. Some data show that the largest market for WCO is in the USA [4]. For Europe, the average amount of WCO per person per year was estimated to be within the range of 1.6–6.5 kg [5], despite the fact that the domestic generation of WCO is almost twice as low compared to the HORECA sector [2]. The relatively easy method of collecting WCO as a separate material, enhanced by increased public consciousness [6], makes it a very attractive source for further sustainable strategies [7]. Part of the oil residues, after a relatively simple treatment, can be used as animal feed [8] or additives for asphalts [9]. However, the majority of WCO is involved in the synthesis of biofuels, which has traditionally been accomplished by transesterification. In the last decade, other thermos–chemical processes for bio-fuels synthesis, such as pyrolysis, hydrocracking and gasification, have gained popularity [2], and their output is considered a value-added product. Another innovative application that takes advantage of the unique structure and relative homogeneity of WCO is represented by bio-lubricants [10,11] and plasticizers [12].

To obtain value-added products that can compete with classical fossil fuel-based solutions (e.g., phthalate plasticizers), further steps, involving chemical modification, are required [3]. The carbon–carbon double bond in the fatty acid residue is the only reactive moiety that can be more conveniently subjected to the epoxidation reaction. Due to the high reactivity of the oxirane ring, the epoxidized oil can be further functionalized to various designed chemicals. Therefore, the epoxidation step plays an important role in the valorization possibilities of WCO. Despite the fact that this reaction has been intensively studied with edible oils and with epoxidized soybean oil (ESO), as a globally available commercial product, its replacement in the industry is still scarce due to high costs [13]. However, the use of WCO, the selectively recyclable catalyst and mild conditions for the epoxidation reaction could overcome this obstacle. For this reason, new publications on oil epoxidation are constantly appearing [14].

On an industrial scale, oil epoxidation is carried out via “in situ”-generated peracids in an acidic homogeneous medium—the so-called Prilezhaev method [15]. H_3_PO_4_, H_2_SO_4_, HCl, HNO_3_ acids are typically used for this purpose, which, along with the need to use large quantities of hydrogen peroxide (H_2_O_2_), raises serious sustainability and safety concerns [16]. In addition, the targeted outcome of the reaction is poor due to several unselective side reactions, such as epoxide ring opening and/or further functionalization [17]. On the other hand, heterogeneous catalytic systems have been developed involving the use of ion exchange resins, phosphotungstate heteropolyacids [17,18], different heterogeneous metal-based catalytic systems [19], including Metal–Organic Frameworks [20] etc. Unfortunately, general drawbacks associated with heterogeneous catalysts such as lower selectivity, mass-transfer limitation and sometimes poor stability issues are also valid.

Enzymatic route is an alternative method that allows for the performance of oil epoxidation with high selectivity in epoxy functional groups [21,22]. In this context, the immobilized lipase from *Candida antarctica B*, commercially available as Novozyme 435, is an example of such biocatalyst [23]. In addition to high selectivity towards the epoxidation reaction, Novozyme 435 has been extensively studied in the epoxidation reaction of different types of oils such as sunflower [24], soybean [25], linseed [26], corn [27] oils or fatty acids [28]. The enzymatic approach allows for better control of peroxy acid formation, requiring mild reaction conditions (e.g., low temperature, ambient pressure and aqueous medium of reaction) and ensuring low toxicity of the whole process. Unfortunately, lipases can also catalyze the hydrolysis reaction of triglycerides, which are the main components of the oil matrix. This undesirable process can reduce the quality of the final product by generating epoxidized mono-, diglycerides and free fatty acids. The addition of toluene and/or fatty acids with long carbon chains (e.g., oleic acid and stearic acid) improves the mass/heat transfer and avoids the hydrolysis of triglycerides [29,30]. Improved enzymatic alternatives for oil epoxidation were recently reported, demonstrating the interest of the research community in this direction [21,22,31]. Notwithstanding all the efforts to provide an optimized green enzymatic process for oil epoxidation, possible industrial applications have not yet been reported. Moreover, to the best of our knowledge, the enzymatic epoxidation of WCO has not been investigated in detail.

In this context, we report a valuable strategy for the enzymatic epoxidation of WCO using a heterogeneous lipase biocatalyst. The sample oil was epoxidized using H_2_O_2_ and a peracid precursor system catalyzed by immobilized lipase (Novozyme 435, NZ). Five different sources of oils were considered for this study (olive, milk thistle, hemp, linseed and sunflower oil), evaluating their composition and also phyco-chemical properties in comparison with those reported in the literature. WCO samples were prepared based on two different approaches (simulated adulteration and real deep-frying process). The optimization of the biocatalytic system was performed using SFw (waste cooking oil from sunflower biomass). System versatility was tested for all the WCOs prepared in this study. The monitorization of the epoxidation process was achieved by comparing the physico-chemical parameters of the WCO before and after epoxidation. The reusability of the enzymatic epoxidation system was evaluated for three successive reaction cycles in the case of SFw. Different strategies for improving system recyclability were considered by looking specifically for the limitation of enzyme leaching, coupled with the good recovery of the catalytic activity of the NZ biocatalyst.

## 2. Materials and Methods

All used chemical reagents of analytical grade were purchased from Sigma-Aldrich (St. Louis, MO, USA). Novozyme 435 biocatalyst (NZ) was purchased from Sigma-Aldrich (Lipase immobilized on acrylic resin, ≥5000 U/g, recombinant, expressed in *Aspergillus niger*).

Vegetable oils (olive, milk thistle, hemp, linseed and sunflower oil) were purchased from the local market. To simulate the adulteration of the oils (SFw), 50 mL of each sample was heated in porcelain crucibles in an oven at 200 °C for 12 h [32,33]. The sunflower waste cooking oil under real conditions (SFr) was collected after deep frying. The solid particles were filtered, and the SFw/SFr was stored in an amber bottle.

### 2.1. NMR Analysis

NMR analysis (Nuclear Magnetic Resonance Spectroscopy (^1^H, ^13^C)) of the oil samples was performed with the Instrument Bruker AvanceIII equipment (Bruker, Carteret, NJ, USA), using CDCl_3_ as a deuterated solvent, at a frequency of 500 MHz. The fatty acid composition of the oils was estimated from NMR spectra according to the reported method [34].

### 2.2. HPLC Analysis

Analysis of the oil samples was performed using a liquid chromatography system HPLC-DAD/RID (Agilent Technologies 1260, Agilent, Stevens Creek, CA, USA). The experimental conditions were as follows: Europhore 100-5 column (L × d = 250 × 4 mm), filled with 5 µm diameter dimethyl-n-octadecylsilane (C18) particles as the stationary phase. The mobile phase consisted of a solvent mixture of ACN:acetone = 41:59 (*v*:*v*), with a flow rate of 1 mL/min, an injection volume of 25 µL and a run time of 30 min. Detection was performed with DAD (210 nm) and RID detectors. The analyzed oil samples were mixed with the mobile phase until complete dissolution without further treatment. 

### 2.3. FTIR-ATR Characterization

FTIR (Fourier Transformed Infrared Absorption Spectroscopy) spectra were obtained using a Perkin Elmer spectrometer, Hamburg, Germany with an ATR (Attenuated Total Reflection) cell equipped with a diamond crystal, in the spectral range of 4000–400 cm^−1^, with a resolution of 4 cm^−1^ and a number of 32 scans, at room temperature. The oil samples were analyzed directly, without any pretreatment, by placing the liquid sample on the crystal and recording the spectrum. After each measurement, the crystal was cleaned with acetone and ethanol to remove all the traces of the measured oil. For the estimation of the unsaturation degree of the sample, FTIR-ATR spectra were subsequently processed using Origin 8.5 software. Spectra were deconvoluted using a Lorenz function, after an initial base line correction [35]. 

### 2.4. Physico-Chemical Characterization of Oil Samples

To estimate the physico-chemical properties of the oils, several indices were determined, as indicated below.

Determination of Saponification Value (SV)

SV represents the amount in mg of KOH required for the saponification of 1 g of oil, and it is correlated with the average molecular weight of triglycerides. Between 1–2 g of oil were refluxed with 25 mL of an alcoholic potassium hydroxide solution for one hour, with continuous stirring. The alkaline excess was titrated with hydrochloric acid (0.5 N) and 1 mL of a phenolphthalein indicator [36]. The phenolphthalein indicator was prepared by dissolving 1 g phenolphthalein in 100 mL ethanol (neutral to phenolphthalein).

SV was calculated using Equation (1), where B = volume in mL of HCl used for the blank test (without oil), S = volume in mL of HCl used for the sample, N = normal concentration of HCl, W = weight of the oil sample (g).

SV = 56.1 × (B − S)N/W(1)

Determination of Acid Value (AV) [37]

AV represents the amount in mg of KOH required to neutralize the free fatty acids in 1 g of oil sample. An amount of 0.1 g to 1.0 g of an oil sample was dissolved in 10 mL of ethanol and ethyl ether mixture (volume ratio 1:1 *v*/*v*). This solution was titrated with a 0.1 N KOH ethanolic solution until the pink color point was reached. Phenolphthalein was used as an indicator. AV was calculated using Equation (2), where V = volume in mL of KOH solution, N = normal concentration of KOH solution, W = weight of the oil (g).

AV = 56.1 × V × N/W(2)

Determination of Peroxide Value (PV)

The PV is a measure of the content of peroxide and other oxidizing species in a certain amount of oil, which oxidizes potassium iodide and releases iodine. The PV is a characteristic of oils’ quality. An amount of 1 g of oil was mixed with 20 mL of chlorophorm:glacial acetic acid = 2:1 (*v*/*v*) and 1 g KI. After 30 min in a dark place, 50 mL of water was added. Finally, the excess iodine was titrated with Na_2_S_2_O_3_ 0.05 N in the presence of the starch indicator. Thiosulfate solution (0.1 N) was prepared from Na_2_S_2_O_3_·5H_2_O crystals in bidistilled water. The solution was standardized with a KIO_3_ solution 0.1 N and 2 g of KI solid in the presence of diluted HCl. A starch indicator solution 1% (*w*/*v*) was prepared by dissolving starch in 10 mL of bidistilled water. The solution was added to 100 mL of boiled bidistilled water, boiled and stirred for three minutes. The resulting solution was cooled and the clear supernatant decanted.

The PV was calculated using Equation (3), where B = volume (mL) of Na_2_S_2_O_3_ used for the blank, S = volume (mL) of Na_2_S_2_O_3_ used for the sample, N = normal concentration of Na_2_S_2_O_3_ solution, W = weight of the oil sample (g).

PV = 1000 × (B − S) × N/W(3)

Determination of Iodine Value (IV) [38] 

The IV represents the degree of unsaturation of the oils, which is expressed in grams of absorbed iodine per 100 g of product. An amount of 0.1–0.2 g of the sample, in accordance with the expected iodine number, was placed into an Erlenmeyer flask. Wijs’ reagent (0.2 N) was prepared from commercial Wijs solution (iodine monochloride) in glacial acetic acid and stored in an amber bottle [38]. An amount of 25 mL of Wijs’ reagent was mixed with 15 mL of chloroform. Chloroform was used instead of carbon tetrachloride, as stated in the official method. After 30 min in the dark, 20 mL of 10% *w*/*v* and 100 mL of bidistilled water were added. Free iodine was titrated with 0.1 N Na_2_S_2_O_3_ solution, using starch solution as indicator. 

The IV was calculated using Equation (4), where B = volume (mL) of Na_2_S_2_O_3_ used for the blank, S = volume (mL) of Na_2_S_2_O_3_ used for the sample, N = normal concentration of Na_2_S_2_O_3_ solution, W = weight (g) of the oil sample.

IV = 12.69 × (B − S) × N/W(4)

The IV(%)—IV conversion was also calculated as follows:

IV(%) = IVfinal × 100/IVinitial − IVfinal)(5)

Determination of the Oxirane Oxygen Content (OOC)

The OOC was measured in accordance with the titration method ASTM D1652-11 and represents the content of oxygen (g) in 100 g of oil [39].

About 0.4 g of used oil or epoxidized used oil was placed in an Erlenmeyer beaker and dissolved in 15 mL of dichloromethane. An amount of 10 mL of a tetraethylammonium bromide (TEAB) solution in acetic acid (100 g in 400 mL) was added to the mixture. Before starting the titration, 5 drops of a crystal violet indicator solution (0.1 g in 100 mL acetic acid) were added to the beaker under magnetic stirring. The titration was performed with the standard solution of 0.1 N perchloric acid in acetic acid until the color changed to green. The crystal violet indicator solution was prepared by dissolving 0.1 g of crystal violet in 100 mL glacial acetic acid. The TEAB solution was prepared from anhydrous tetraethylammonium bromide in glacial acetic acid (100 g in 100 mL).

Th OOC was calculated according to Equation (6), where N = normal concentration of the perchloric acid solution, V = volume used by the perchloric acid solution (mL), and W = weight (g) of the oil sample.

OOC = (1.6 × N × V)/W(6)

OOC theoretic (OOC_teor_) was calculated according to Equation (7), where Ai = 126.9 and Ao = 16.0 are the atomic masses for iodine and oxygen, respectively, IVo is the iodine value (IV) for the initial sample of oil, before epoxidation.

OOC_teor_ = {(IVo/2Ai)/[100 + (IVo/2Ai)Ao]} × Ao × 100 (7)

Relative conversion to oxirane (RCO %) was calculated using the relationship (8), where OOC_exp_ is the experimental oxiranic oxygen content [40,41].

RCO = (OOC_exp_/OOC_teor_) × 100 (8)

### 2.5. Synthesis of NZ-GA Biocatalyst

Modification of the NZ with glutaraldehyde (GA) was performed following the protocol reported previously [42], with a slight modification. An amount of 50 mg NZ was mildly stirred with a solution of 0.25% (*v*/*v*) GA (prepared from 25% (*v*/*v*)) in a PBS buffer (0.5 M, pH 7) at a temperature of 30 °C for 4 h. The solid was filtered and washed several times with buffer and water.

### 2.6. Synthesis of NZ-TEOS Biocatalyst

NZ covering with silica was performed as follows: to a suspension containing 50 mg of NZ suspended in 1 mL H_2_O (A), solution B, containing 50 μL of (tetraethyl orthosilicate) TEOS and 7.5 μL of ((3-aminopropyl)triethoxysilane) APTES, was introduced dropwise under stirring. The suspension was stirred for 4 h at 30 °C. The solid NZ-TEOS was separated by centrifugation from the slurry and washed several times with water.

### 2.7. Determination of the Biocatalyst Activity

The lipase activity was determined based on p-nitrophenylbutyrate (p-NPB) method [43]. An amount of 1 mg of biocatalyst catalyzed in time the hydrolysis of 50 µL of 1 mM p-NPB solution in ACN. A p-NPB solution was added to 2.95 mL of a solution containing H_2_O:Tris-HCl buffer 50 mM (with pH-7.5) in a 59:1 (*v*/*v*) ratio at 25 °C. One international unit of activity (U) was defined as the amount of enzyme that hydrolyzes 1 μmol of p-NPB per minute under the described conditions. 

### 2.8. Determination of the Enzyme Leaching

Enzyme leaching was performed based on the approach reported previously in the literature [44]. Solid biocatalysts (NZ, NZ-GA and NZ-TEOS) were mixed with dimethylsulfoxyde (DMSO) (1/100 *w*/*v*) and stirred for 1 h at 37 °C. The amount of extracted enzyme in DMSO was determined using the Coomassie (Bradford) Protein Assay method [45] by measuring the change in absorbance at 595 nm and using lipase from *Aspergillus niger* as the reference. The amount of leached enzyme was expressed as mg of enzyme per g of biocatalyst.

### 2.9. The Procedure for the Enzymatic Epoxidation of Oils Sample 

All the tests were performed in 5 mL glass vials with a screw cap. An amount of 2 g of the oil sample was introduced in vials together with 0.5–2.5 wt% of the biocatalyst (NZ/NZ-GA/NZ-TEOS). For each type of oil, the appropriate amount of 30% H_2_O_2_ and peracid agent was added to ensure the intended molar ratio between the components, based on the concentration of double bonds in each sample. The magnetic stirring and heating were achieved by a magnetic stirrer hot plate equipped with a thermocouple and a metal block. Once the reaction time had elapsed, vials were cooled and centrifuged. The formation of three phases was observed: the water phase at the bottom, solid soap and enzyme in the middle and the oil phase at the top. Only the oil phase was removed for further analysis. The biocatalyst was washed several times with water and air-dried before the recyclability tests were performed.

### 2.10. Data Representation

All the samples for the experimental study were prepared in three replicates. In addition, the analysis of the physico-chemical characterization of the oil samples and the determination of the enzyme activity/leaching were performed in triplicate. The mean value and standard deviation were calculated based on the corresponding replicates and considered for preparing the figures and tables.

## 3. Results and Discussion

### 3.1. WCO Characterization

Five sources of vegetable oils (Table 1) were considered for studying the correlation between the efficiency of the enzymatic epoxidation process and the compositions of the oil, looking specifically for the (un)saturation degree of the fatty acids residue and other physico-chemical parameters such as saponification, acidity and peroxide values. The five sources of oils and the corresponding abbreviations are listed below, in Table 1. Additionally, the content of the fatty acid residue based on the literature reports is also listed, offering a perspective of the corresponding WCO content. 

The fatty acid profile of the oil samples represents important information regarding the ethylenic equivalent of these samples (saturated/unsaturated degree), which is relevant for further epoxidation. The degree of unsaturation (expressed as iodine value IV) is therefore a valuable parameter for the epoxidation process, as it limits the amount of H_2_O_2_ required as oxidation reagent. According to the literature (Table 1), the OL oil is the richest in monounsaturated fatty acids (64.4–72.7% oleic acid), while the HM and LS oils contain higher amounts of highly multi-unsaturated fatty acids (50–70% linoleic acid and 49–56% linolenic acid, respectively). 

The NMR analysis (especially ^1^H NMR) was performed to confirm the composition of the oils reported in the literature (Table 2). It allows a non-destructive and fast analysis of samples providing the fatty acid profile [34,51,52] and the quantitative assessment of the physico-chemical parameters [53]. In addition, the progress of the enzymatic epoxidation process can be easily followed [54]. The composition of the oils in terms of fatty acids was estimated from the NMR spectra, according to the reported method [34]. The experimental results are resumed in Table 2. It is obvious that the ^1^H NMR distribution of fatty acids is in agreement with that reported in the literature (Table 1).

For all five vegetable oils, the physico-chemical parameters, such as saponification (SV), iodine (IV), acidity (AV) and peroxide (PV) values, were measured for both fresh and WCO alternatives. The results are presented in Table 3. For fresh oils, the unsaturation degree, represented by the iodine value (IV) varied in the following order: OL < MT < SF < HM < LS. In addition, the acidity and peroxide values exceeded the accepted limits for almost all samples [55].

For the WCO, the saponification value (SV) was high, indicating a lower average molecular weight of the fatty acid residues compared to the oil sources. In addition, high values were obtained for PV, which is a common direct effect of the thermal treatment of the oil samples [47]. The PV increased significantly in the case of SFw and HMw, and it was almost doubled for the WCO (SFr) subjected to deep-frying. The OLw, MTw and LSw oils were the least subjected to oxidation, judging by the difference in the PV values. 

**Figure 1:**
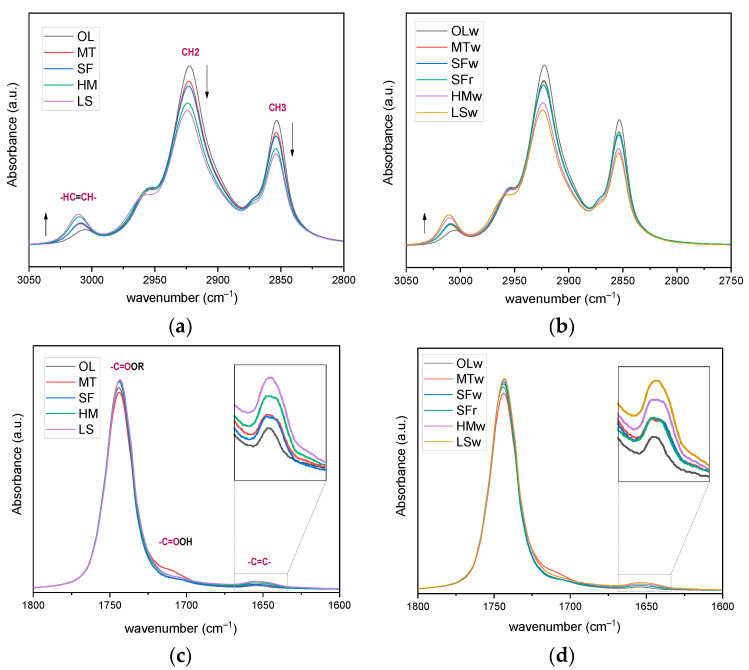
FTIR-ATR spectra recorded for fresh (**left**) and wasted (**right**) oils for 1600–1900 cm^−1^ (**a**,**b**) and 2800–3050 cm^−1^ (**c**,**d**) regions.

The composition of the WCO samples was evaluated using IR spectrophotometry, considering the corresponding fresh oil as a reference. FTIR-ATR spectra are presented in Figure 1. The main absorption bands in the IR spectra were assigned based on the data reported in the literature [35]. Two spectra regions are significantly related to the sample composition (1600–1900 cm^−1^ and 2800–3050 cm^−1^). In the region 1600–1900 cm^−1^, there are three bands specific to the stretching vibrations: –C=C– (cis) groups at 1653 cm^−1^ (Figure 1a,b), the carbonyl –C=O in esters and free acids at 1743 cm^−1^ and 1709 cm^−1^, respectively [56]. The fresh MT oil contains more free fatty acids, which can be also observed from the ATR spectra at 1710 cm^−1^ (Figure 1c) and from the higher AV (Table 3).

In the region 2800–3050 cm^−1^ (Figure 1c,d), there are significant vibrations of the =C–H groups (more intense cis and trans), originating from triglyceride fractions at the 3006–3010 cm^−1^ bands at 2919 and 2846 cm^−1^ for the –C–H stretching vibrations of the CH_2_ and CH_3_ groups in the aliphatic chain of triglycerides [57]. The WCO samples show only a slight difference compared to the fresh ones. As can be observed, the intensity of the bands at 3006–3010 cm^−1^ (=C–H) and 1653 cm^−1^ (–C=C–) increases from OL to LS in fresh oil (Figure 1c) but also in WCO (Figure 1d). 

**Figure 2:**
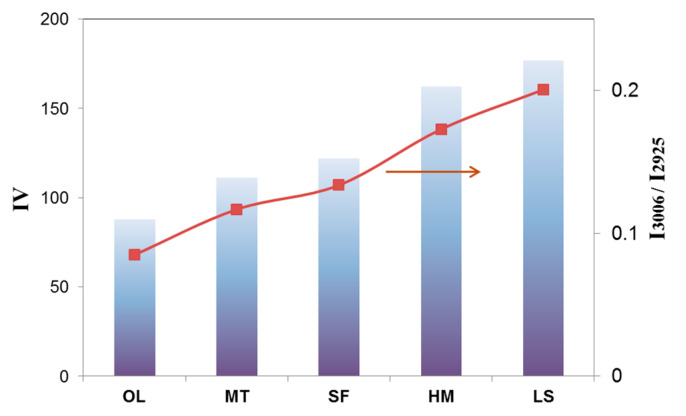
Unsaturation degree of the fresh oils evaluated as IV, mean values (columns) and I3006/I2925 ratio based FTIR-ATR analysis (red line) (RSD < 20%).

Figure 2 represents the IV of the fresh oils. Additionally, IV, estimated by FTIR-ATR analysis using the ratio of the band intensities A3006/A2925, as previously reported [58], shows the same unsaturation trend as reported in the literature (Table 3). A lower IV suggests lower unsaturation. 

An HPLC analysis was also used for the evaluation of the mono-, di- and triglycerides (MG, DG, TG), as well as of the free fatty acids composition for WCO and corresponding fresh oils. The results are consistent with those shown in Table 1 and Table 2. A high content of linoleic acid, followed by oleic and palmitic acids, mostly present as triglyceride components, was confirmed for MT and SF. Stearic acid was present in small amounts [59]. Triglycerides with a high linolenic acid content were detected for HM and LS [60]. The OL oil has a unique feature. As the most saturated oil, it is rich in triglycerides containing monosaturated and unsaturated fatty acids. In the chromatograms of the WCO, the formation of mono- and diglycerides was observed. It is interesting to note that SFr has a similar content as fresh SF oil.

### 3.2. Setup Optimum Conditions of the Enzymatic Epoxidation Process

**Scheme 1:**
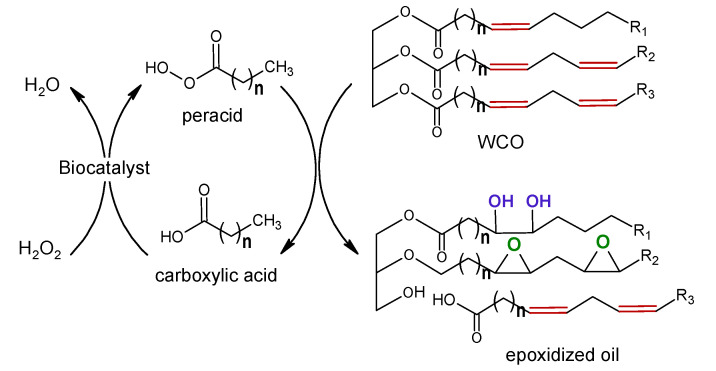
Enzymatic epoxidation with “in situ” formation of peracid.

The system of the enzymatic epoxidation of oil samples has been developed, looking specifically for the WCO(w/r) substrate. The unsaturated fatty acid chain is attacked by the peroxy acid generated previously “in situ” with an added carboxylic acid (e.g., formic, propanoic, butanoic and octanoic acid, etc.). The lipase enzyme, designed as the Novozyme type (NZ), catalyzed the peroxy acid production using H_2_O_2_ as oxidation reagent (Scheme 1).

The optimization of the enzymatic epoxidation system was performed testing the effects of H_2_O_2_ and biocatalyst concentration, the type and concentration of carboxylic acid used as a precursor of peroxy acid and also the reaction time of the biocatalytic epoxidation process. The optimum values of the tested parameters were set up by evaluating the system’s performance, expressed as iodine value conversion IV(%), conversion to oxirane RCO and sample acidity AV. The optimization of the enzymatic process was performed on SFr as the reference system. The concentration of oil was maintained constant during these experiments, while all the other reactants were adjusted to improve the performance of the epoxidation process correspondingly.

**Figure 3:**
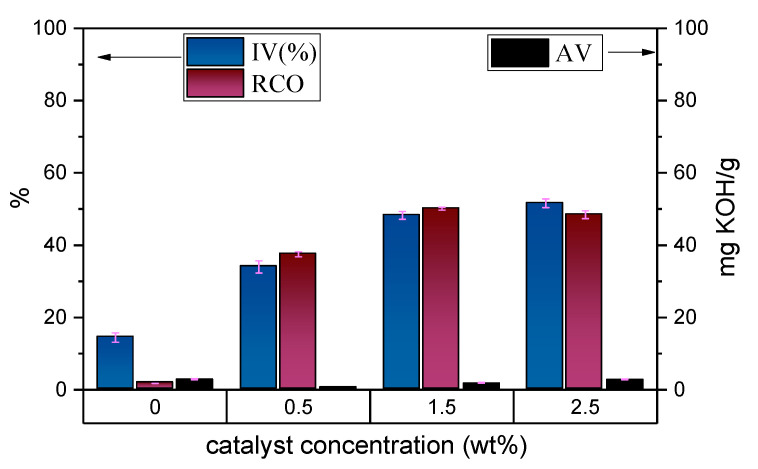
The influence of the NZ concentration on the bio-epoxidation process. Experimental conditions: 2 g of SFr oil; H_2_O_2_ and acid concentration of 3 mM; temperature of 30 °C; 6 h reaction time.

Different concentrations of the enzyme catalyst (NZ) were tested in the proposed system. The epoxidation results correlated with the biocatalyst concentration are shown in Figure 3. It can be observed that increasing the loading of the biocatalyst from 1.5 wt% to 2.5 wt% led to the increased acidity of the samples, without significant changes in the double bond epoxidation. Therefore, the 1.5 wt% NZ was further used. This is in accordance with the literature data (1–7 wt% of the enzyme catalyst for biocatalytic epoxidation of oleic acid and tall oil) [28]. 

**Figure 4:**
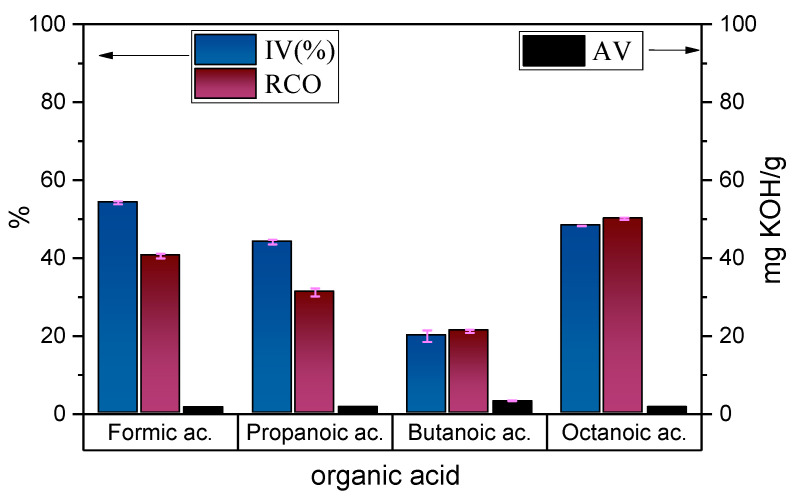
The influence of the peracid agent on the bio-epoxidation process. Experimental conditions: 2 g SFr; 1.5 wt% NZ, H_2_O_2_ and acid concentration: 3 mM; temperature of 30 °C; 6 h reaction time.

Four different organic acids (formic, propanoic, butanoic and octanoic acids) were tested for the biocatalytic epoxidation of the SFr sample. The results are summarized in Figure 4. In the presence of formic acid, more than 50% of the ethylenic bonds are converted (54.2 IV(%) and 40.6 RCO(%)). Despite the good result obtained in the presence of formic acid and the advantage of its easier elimination from the reaction mixture, a significant build-up pressure was observed due to the decomposition of formic acid in CO_2_. The difference of about 10% between the IV(%) and RCO(%) values also suggests that further side reactions occurred at the formed epoxide. Therefore, alternative acids, such as propionic, butyric and octanoic acids, were tested, too (Figure 4). The experimental results demonstrated that the increase in the length of the acid chain led to an increase in the conversion of the epoxidation process. Octanoic acid allowed for obtaining the maximum conversion for the sample epoxidation (48.3 IV(%), 50.1 RCO(%)), without modifying the system pressure, as noticed before with formic acid. This confirms that the octanoic acid can be successfully used as an “in situ” peracid source in the biocatalytic system of the epoxidation process. Similar results were previously reported in the literature [31].

**Figure 5:**
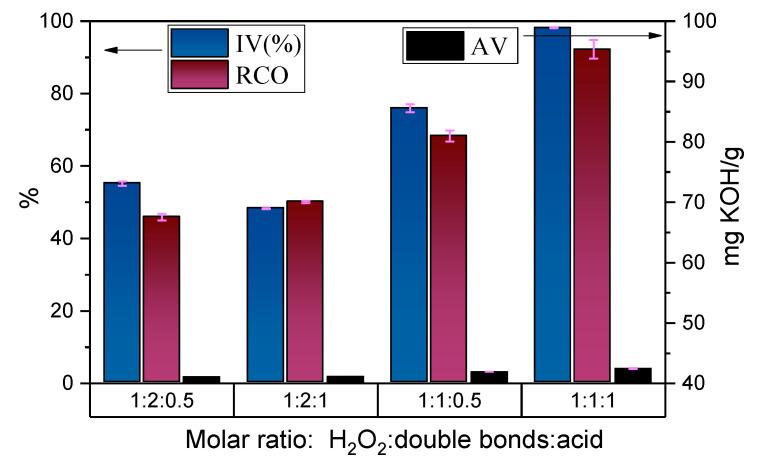
Screening of the ratios of H_2_O_2_ to double bonds and peracid agent. Experimental conditions: 2 g SFr, 1.5 wt% NZ; 30 °C temperature, 6 h reaction time.

The performance of the biocatalytic system was evaluated by varying the molar ratio of H_2_O_2_/double bonds/octanoic acid. The experimental results are presented in Figure 5. At a lower H_2_O_2_ concentration, the double bond conversion to epoxide was lower, without any visible influence of the peracid agent concentration. Almost complete epoxidation of the sample was obtained for 1:1:1 = H_2_O_2_/double bonds/octanoic acid (molar ratio). Usually, the molar ratio of H_2_O_2_ to double bonds used is 1–2 [28]. On the other hand, the peroxide is mostly responsible for the deactivation of the enzyme catalyst and the problem related to the generated high pressure. In this study, the amount of H_2_O_2_ was kept as low as possible, not exceeding the H_2_O_2_/double bonds ratio of 1. It is well known that a high concentration of octanoic acid can increase the initial rate of the reaction [61]. However, because octanoic acid is the substrate that binds first to the enzyme, using a high amount of acid can also inhibit the biocatalyst. Additionally, some of the epoxide is lost when the high excess of the octanoic acid has to be removed from the system (washing step [61]). For this reason, a 1:0.5 ratio of H_2_O_2_ and octanoic acid was set up as optimum conditions.

The most important results of the initial screening for the SFr epoxidation experiments are resumed in Table S1 (Supplementary Materials section).

**Figure 6:**
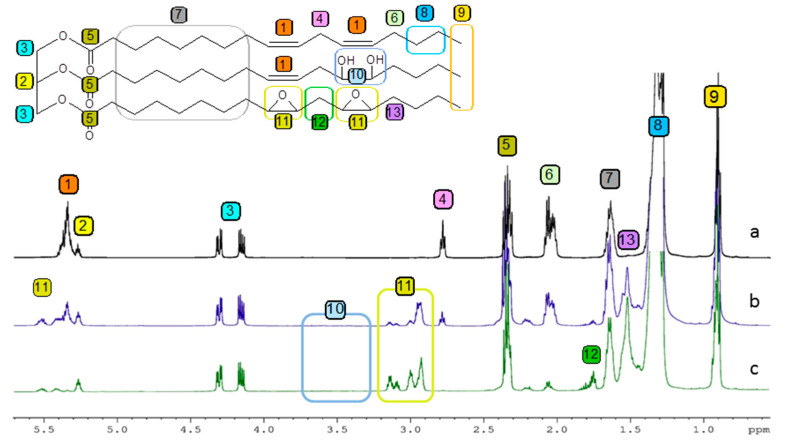
^1^H NMR spectra of (a) SFr and epoxidized SFr for (b) 6 h and (c) 12 h. Experimental conditions: 2 g SFr; 1.5 wt% NZ; molar ratio H_2_O_2_/double bonds/acid—1:1:0.5; temperature of 30 °C.

**Figure 7:**
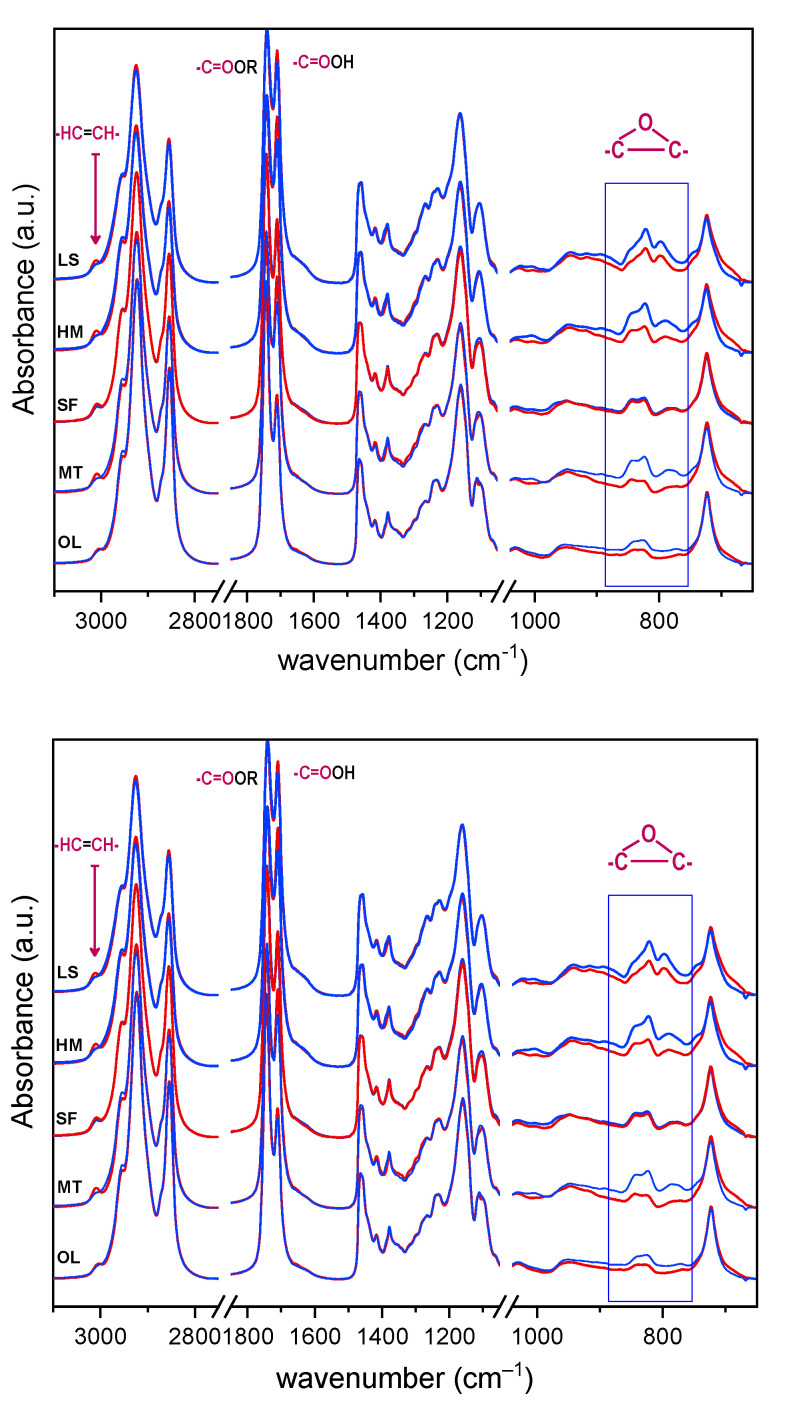
FTIR-ATR spectra for epoxidized fresh oils (blue line) and wasted oils (red line). Experimental conditions of the enzymatic epoxidation process: 2 g oil, 1.5 wt% NZ, molar ratio H_2_O_2_/double bonds/acid—1:1:0.5; temperature of 30 °C and 6 h reaction time.

**Figure 8:**
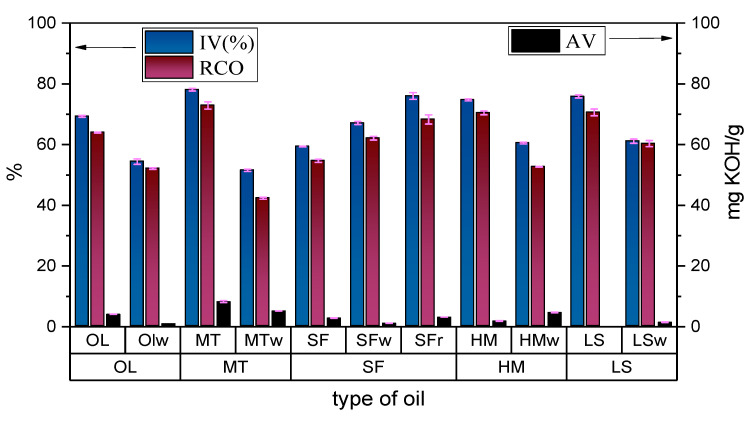
Enzymatic epoxidation of fresh and wasted oils. Experimental conditions: 2 g oil, 1.5 wt% NZ, molar ratio H_2_O_2_/double bonds/acid—1:1:0.5; temperature of 30 °C and 6 h reaction time.

**Figure 9:**
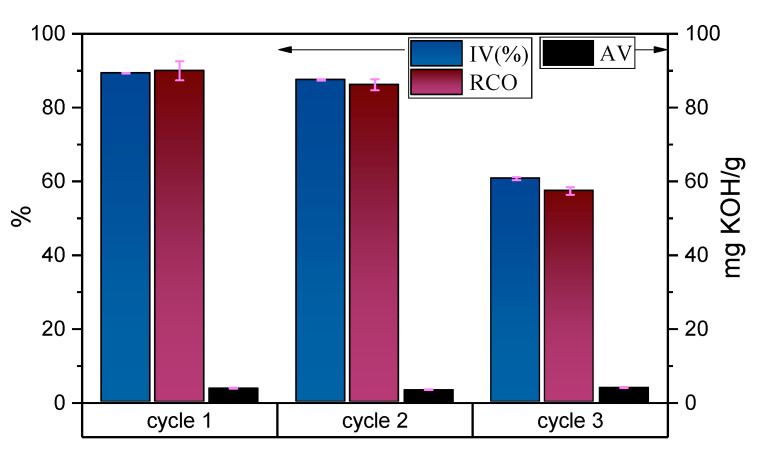
Testing the recyclability of the setup enzymatic epoxidation system. Experimental conditions: 2 g SFr, 1.5 wt% NZ, molar ratio H_2_O_2_/double bonds/acid—1:1:0.5; temperature of 30 °C and 12 h reaction time.

**Figure 10:**
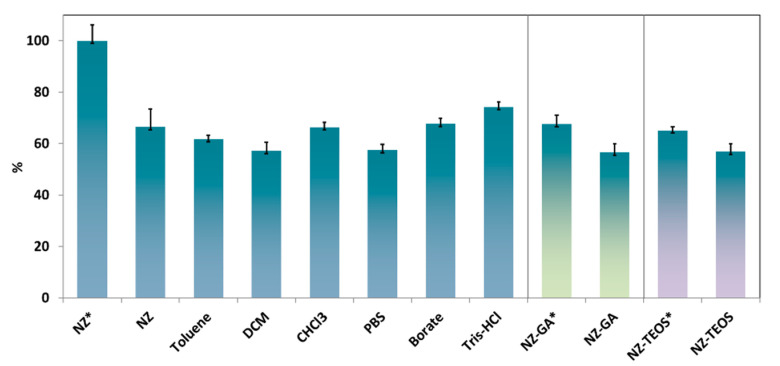
Recovery of the enzymatic activity after three reaction cycles (the initial NZ activity was considered as reference). Experimental conditions: 2 g SFr, 1.5 wt% catalyst, molar ratio H_2_O_2_/double bonds/acid—1:1:0.5. When an organic solvent or buffer was used, 0.5 mL of the corresponding liquid was additionally added; temperature of 30 °C and 12 h reaction time. *—fresh catalyst.

**Figure 11:**
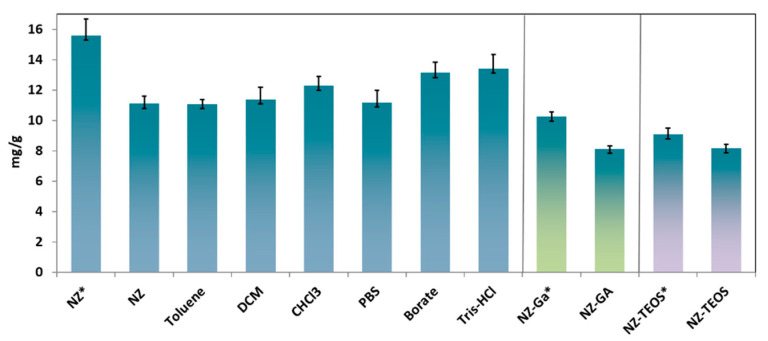
Improving the enzyme loading of the biocatalyst. Experimental conditions: 2 g SFr, 1.5 wt% catalyst, molar ratio H_2_O_2_/double bonds/acid—1:1:0.5. An amount of 0.5 mL of the corresponding liquid was additionally added; temperature of 30 °C and 12 h reaction time. *—fresh catalyst.

The enzymatic epoxidation of SFr was performed for two different reaction times (6 h and 12 h). The ^1^H NMR analysis of the samples after epoxidation allowed for the monitoring of the epoxidation process (Figure 6). The intensity of specific signals at 5.3 ppm (1), corresponding to protons of double bonds and protons of CH_2_ groups (4, 6), situated between and next to double bonds, decreases during the epoxidation reaction (Figure 6a–c). At the same time, new signals, specific for protons of the epoxide ring in the range of 2.9–3.2 ppm (Figure 6b,c—(11)), together with new signals of CH_2_ group between and adjacent to epoxides (Figure 6b,c—(12, 13)) confirm a successful epoxidation. Moreover, no signals (10) attesting to epoxide ring opening and hydroxyl formation were detected. A slight decrease in the signals corresponding to the protons from the glycerol chain (2, 3) suggests a partial hydrolysis of glycerides to free fatty acids (Figure 6b,c) [45]. In conclusion, 12 h is the best reaction time in terms of achieving performant epoxidation of the sample and minimizing undesirable reactions, such as oxirane ring opening and hydrolysis of triglycerides. 

Additionally, a FTIR-ATR analysis was also performed. In the FTIR-ATR spectra presented in Figure S1, the formation of oxirane rings in time was noticed in the range 800–860 cm^−1^. 

The ^1^H NMR results were also confirmed using the HPLC analysis method (Figure S2). The epoxidized mono-, di- or triglycerides were detected in the reacted mixture [62,63].

### 3.3. Enzymatic Epoxidation of WCO

The WCOs characterized in the previous experiments (Section 3.1) were considered for enzymatic epoxidation. In addition, the corresponding fresh oils were enzymatically epoxidized for comparison. The bio-epoxidation system was setup according to the optimization steps described previously (Section 3.2). The epoxidized samples were analyzed using the FTIR-ATR method. FTIR-ATR spectra are presented in Figure 7. The epoxide species were identified in the spectra of both fresh and waste oils after epoxidation. Higher intensities of the bands in the range 760–860 cm^−1^, corresponding to the oxirane ring vibrations, were clearly observed.

The system performance was evaluated by calculating the IV(%), RCO(%) and AV and comparing with similar characteristics of the oil samples before bio-epoxidation. The experimental results are shown in Figure 8 and Table S2. Generally, the WCO samples exhibited lower IV(%) after epoxidation compared to fresh oils (Figure 8). Additionally, a significant decrease in the IV was noticed after the bio-epoxidation process (Table S2). This provides clear evidence of the efficient epoxidation of all the samples. The epoxidation of SFw and SFr samples was particularly efficient. In this case, a high content of free fatty acids, which served as sources of peracid in the initial samples, is the main reason for this behavior. These conclusions are also supported by the FTIR-ATR results (Figure 7). In addition, high RCO(%) values for all the epoxidized samples (within the range of 42 and 75%) confirm the success of the biocatalytic process. The differences in RCO among the tested samples are due to the correlation between the physico-chemical characteristics and the fatty acids composition of the corresponding oils. Other factors, such as viscosity and density of the oil samples before and after epoxidation, together with additional non-lipid components and impurities may also influence the rate of the epoxidation reaction [64]. Such impurities may include ketones, aldehydes or other products of oil oxidation, polycyclic aromatic hydrocarbons, polychlorinated biphenyls, acrylamides, dioxin-like compounds, etc. [65,66,67]. Their presence depends on the type of oil, the food being fried and the frying conditions [67]. The contaminants can bind to the surface of the catalysts and slow down the catalytic activity [65].

The AV did not exhibit any significant variations after the epoxidation process (Table S2). Therefore, the epoxidation step was achieved preponderantly against the hydrolysis step. Moreover, the AVs of the initial samples can, to some extent, be correlated with the success of the epoxidation process because a high content of free fatty acids provides a source of peracid species. The behavior of the MT sample is an notable example. However, the saturation grade of fatty acids in OLw and LSw did not affect the epoxy selectivity. 

### 3.4. Recyclability of the Enzymatic Epoxidation System

The enzymatic epoxidation of SFr was performed for three times using the same NZ biocatalyst in order to evaluate the system’s recyclability. The experimental results are presented in Figure 9. For the first two cycles, the IV(%) and the RCO(%) value did not show significant differences. However, both parameters decreased in the third cycle. The AV was preserved during the experiments.

The temperature of 30 °C, even for a longer reaction time (12 h), allowed for the achievement of almost 90% conversion, and this result was maintained for two catalytic cycles. 

As can be observed, the system efficiency started to decrease during the third reaction cycle. The main reason was the deactivation of the lipase enzyme. This was a direct effect of enzyme denaturation due to temperature, H_2_O_2_ presence and also enzyme leaching [17,23,68,69].

To improve the system’s reusability, a high recovery of enzyme activity was required. The main factors leading to this deactivation of the lipase during the epoxidation reaction are the high concentration or excess of H_2_O_2_ or even the presence of the epoxide and oxygenated by-products [23,68,69]. In this context, the biocatalytic tests were carried out with the addition of organic solvents (toluene, DMC or CHCl_3_) and buffer solutions (PBS, borate or Tris-HCl) to reduce the contact of the lipase enzyme with H_2_O_2_ [30,68]. The addition of the organic solvent offers the mandatory hydrophobicity for lipase activation and a proper environment for the preservation of the peroxy-acid species [30,70]. The buffer addition inhibits the opening of the epoxide ring and more effectively removes the residual soaps during washing [71].

Additionally, various coating and crosslinking approaches can be used for limiting enzyme leaching to improve the biocatalyst’s reusability [17,42,72]. In addition, the NZ biocatalyst was redesigned for a similar reason, by covering it with (glutaraldehyde) GA or (tetraethyl orthosilicate) TEOS, leading to the NZ-GA and NZ-TEOS biocatalysts, respectively [17,42,72].

The experimental results were compared with those obtained previously, focusing on enzyme leaching and the recovery of the biocatalyst activity after three successive reaction cycles (Figure 10 and Figure 11). 

Enzyme leaching was also investigated for the NZ and also for the NZ-GA and NZ-TEOS biocatalysts. The results are presented in Figure 11. The enzyme loading of the NZ biocatalyst after three reaction cycles was 12.3, 13.1 and 13.4 mg/g enzyme in the case of CHCl_3_, borate and Tris-HCl addition, respectively. Only 11.1 mg/g enzyme loading was determined when the epoxidation was performed without any solvent/buffer addition. An amount of 15.6 mg/g of enzyme was the initial enzyme loading of NZ. Therefore, the addition of CHCl_3_, borate or Tris-HCl buffer reduced the enzyme leaching of the NZ biocatalyst. For NZ-GA and also for NZ-TEOS, the enzyme loading was better preserved after three reaction cycles compared with NZ. In terms of the enzyme activity (Figure 10), the positive effect of solvent/buffer addition was observed, similar to the behavior described previously for enzyme loading. NZ-GA and NZ-TEOS offered more valuable alternatives for the good recovery of the enzyme biocatalytic activity.

The monitorization of the epoxidation process was achieved based on the FTIR-ATR analysis. The spectra of the epoxidized and initial samples are presented in Figure S3. A better performance of the bio-epoxide system can be observed in the presence of CHCl_3_, borate or Tris-HCl buffer compared with epoxidation without any additional solvent.

## 4. Conclusions

The enzymatic system setup in this study allowed for the performance of biocatalytic epoxidation of WCO with good performance. Therefore, the results obtained in this study contribute to addressing the lack of data regarding the enzymatic epoxidation of WCO. It was confirmed that sunflower WCO (SFw/SFr) can be selectively epoxidized with about 90% conversion in the presence of the NZ biocatalyst at a low temperature (30 °C) and a relatively short reaction time (12 h). All of WCO tested in this study exhibited good conversion to the epoxide. For SF oil, the double abundance of the peroxide species generated during the adulteration of the oil led to modifications occurring during bio-epoxidation. It was demonstrated that octanoic acid, which improves the safety of the epoxidation process, is the best enzyme substrate for peracid formation among other organic acids with shorter chains. The optimum experimental conditions set up for the enzymatic epoxidation of SFr (molar ratio of H_2_O_2_/double bonds/peracid agent = 1:1:0.5, 1.5 wt% NZ and 12 h reaction time) allowed for the production of an epoxidized sample characterized by 89 IV(%), 90 RCO(%) and 4 (mgKOH/g) AV. The applicability of the developed system to five different oil samples (OL, MT, SF, HM and LS) provides clear evidence of the system’s versatility.

For the system’s reusability, both enzyme leaching and the activity recovery of the biocatalyst were considered. Enzyme leaching was reduced by the addition of CHCl_3_, borate or Tris-HCl buffer. However, the use of a buffer is more advantageous due to its easy separation by centrifugation. In addition, covering NZ with GA or TEOS (NZ-GA and NZ-TEOS biocatalysts) reduced enzyme leaching. This is an additional alternative for biocatalyst recyclability that deserves further investigation.

## Figures and Tables

**Table 1:** Reported composition (%) of fatty acids residue in studied oils.

Oil Source	Abbreviation	Fatty Acids (%)				
		Palmitic Acid, C16:0	Stearic Acid, C18:0	Oleic Acid, C18:1	Linoleic Acid, C18:2	Linolenic Acid, C18:3
Olive (*Olea europaea*) extravirgin oil	OL [46]	12–15	2.1–2.7	64.4–72.7	10.3–15.0	0.6–0.9
Milk thistle (*Silybum marianum* (L.) *Gaertn.*) oil	MT [47]	5–7	4–5	15–22	57–60	0–1
Sunflower (*Helianthus annuus*) oil	SF [48]	5–7.6	2.7–6.5	14–49	48.3–74.0	0–0.3
Hemp (*Cannabis sativa*) oil	HM [49]	6–9	2–3	10–16	50–70	15–25
Linseed (*Linum usitatissimum*) oil	LS [50]	4.5–5.5	4–6.5	19.5–28	12.5–15.5	49–56

**Table 2:** Fatty acid profile (%) of studied oils, according to ^1^H NMR analysis.

Fatty Acids (%)	Oil				
	OL	MT	SF	HM	LS
Linolenic ac.	2.0	1.6	0	26.7	48.4
Linoleic ac.	2.6	45.8	42.4	41.2	2.5
Monounsaturated ac.	79.6	33.4	45.1	13.1	30.2
Saturated ac.	15.8	19.2	12.5	19	18.9

**Table 3:** Physico-chemical parameters of the fresh and WCO oils.

Oil	Indices *			
	SV	AV	IV	PV
OL	196.6 ± 3.5	3.4 ± 0.06	88 ± 1.8	10 ± 0.2
Olw **	201.9 ± 2.6	3.6 ± 0.06	84.3 ± 1.0	11.2 ± 0.1
MT	196.7 ± 4.4	9.0 ± 0.15	113.9 ± 2.3	9.9
MTw **	203.3 ± 1.7	9.7 ± 0.06	111.3 ± 2.2	11.2 ± 0.1
SF	198 ± 3.6	2.1 ± 0.1	122 ± 3.0	16 ± 0.2
SFw **	204 ± 3.3	2.1	115.7 ± 0.9	19.5 ± 0.2
SFr	204.1 ± 2.6	2.8	120.7 ± 1.0	34.9 ± 0.9
HM	196.8 ± 5.2	3.7	162.5 ± 4.0	10
HMw **	207 ± 3.5	4.2 ± 0.1	160.9 ± 2.2	19.9 ± 0.5
LS	195 ± 2.1	3.6	177 ± 1.9	5
LSw **	198.7 ± 3.1	4.1 ± 0.1	176.8 ± 4.5	6

* SV—saponification value (mgKOH/g), AV—acidity value (mgKOH/g), IV—iodine value (gI_2_/100 g), PV—peroxide values (meqO_2_/kg). ** The letter “w” was used to denote waste oils obtained after simulated thermal adulteration. The letter “r” was used for the waste oil obtained from deep-frying in real conditions.

## Data Availability

The original contributions presented in the study are included in the article and Supplementary Materials, further inquiries can be directed to the corresponding author.

## Supplementary Materials

The following supporting information can be downloaded at: https://www.mdpi.com/article/10.3390/ma17184518/s1, Table S1: Physico-chemical parameters of the SFr oil after epoxidation and the conversion of SFr oil for the epoxidation process using different experimental conditions. Table S2: Physico-chemical parameters and conversion of fresh and wasted oils. Figure S1: FTIR-ATR spectra for SFr before and after epoxidation. Figure S2: HPLC profile of the SFr after consecutive cycles of enzymatic epoxidation. Figure S3: FTIR-ATR spectra of SFr samples.
