# Supplementary material for: Sustainable Biocatalytic System for the Enzymatic Epoxidation of Waste Cooking Oil
Source: Materials (Basel). 2024 Sep 14;17(18):4518. doi: 10.3390/ma17184518 (PMC11433602; doi:10.3390/ma17184518)
Supplement: Supplementary file 1 [file materials-17-04518-s001.zip › materials-3160254-supplementary.pdf]

Sustainable Biocatalytic System for the Enzymatic Epoxidation of Waste Cooking Oil

Iunia Podolean and Madalina Tudorache *

Department of Organic Chemistry, Biochemistry and Catalysis, Faculty of Chemistry, University of Bucharest, 4-12 Regina Elisabeta Av., 030018 Bucharest, Romania
* Correspondence: madalina.sandulescu@g.unibuc.ro

Supplementary Materials:

Table S1. Physico-chemical parameters of the SFr oil after epoxidation and the conversion of SFr oil for the epoxidation process using different experimental conditions.

Entry	Catalyst (wt%)	H_2O_2/double bonds[a]	Acid[b]	H_2O_2/acid[a]	IV	IV(%)	OOC	RCO(%)	AV
1	0	0.5	O	1	103.3 ± 2.1	14.4 ± 1.3	0.1	1.8 ± 0.03	2.9 ± 0.16
2	0.5	0.5	O	1	79.7 ± 2.3	34.0 ± 1.7	2.7 ± 0.04	37.5 ± 0.7	0.8
3	1.5	0.5	O	1	64.5 ± 0.8	48.3 ± 0.2	3.5 ± 0.06	50.1 ± 0.04	1.9 ± 0.1
4	2.5	0.5	O	1	58.4 ± 1.8	51.6 ± 1.2	3.4 ± 0.09	48.4 ± 1.6	2.9 ± 0.1
5	1.5	0.5	O	1	64.5 ± 1.9	48.3 ± 1.1	3.5 ± 0.05	50.1 ± 0.4	1.9 ± 0.06
6	0	0.5	O	1	103.3 ± 2.5	14.4 ± 1.4	0.1	1.8 ± 0.02	2.9 ± 0.04
7	1.5	0.5	P	1	67.5 ± 1.3	44.1 ± 0.6	2.2 ± 0.06	31.2 ± 1.0	1.9
8	0	0.5	P	1	83.7 ± 1.4	30.7 ± 0.6	1.9 ± 0.02	26.3 ± 0.4	2.4 ± 0.1
9	1.5	0.5	B	1	96.6 ± 2.7	20.0 ± 1.5	1.5 ± 0.02	21.2 ± 0.4	3.4 ± 0.1
10	0	0.5	B	1	112.8 ± 2.8	6.5 ± 1.6	0.6	8.1 ± 0.15	3.0 ± 0.03
11	1.5	0.5	F	1	55.2 ± 0.9	54.2 ± 0.4	2.9 ± 0.04	40.6 ± 0.5	1.9
12	0	0.5	F	1	60.8 ± 1.4	49.6 ± 0.7	2.9 ± 0.02	40.5 ± 0.6	2.0 ± 0.05
13	1.5	0.5	O	2	54.1 ± 1.2	55.2 ± 0.6	3.5 ± 0.05	45.9 ± 0.9	1.8
14	1.5	0.5	O	1	64.5 ± 0.4	48.3 ± 0.1	2.7 ± 0.04	50.1 ± 0.3	1.9
15	1.5	1	O	2	29 ± 1.5	76.0 ± 1.1	4.8 ± 0.07	68.3 ± 1.5	3.2 ± 0.03
16	1.5	1	O	1	4.6 ± 0.2	96.2 ± 0.1	6.5 ± 0.12	92.2 ± 2.6	4.1 ± 0.12

17	0.5	0.5	-	0	111 ± 1.0	8.1 ± 0.4	0.5	7.1 ± 0.1	1.9 ± 0.05
18	0	0.5	F	2	71.9 ± 1.1	39.0 ± 0.4	2.5 ± 0.04	35.2 ± 0.5	3.4 ± 0.1

Reaction conditions: SFr—2g, T—30°C, time—6 h. [a] Molar ratio; [b] O—octanoic acid, P—propanoic acid, B—butanoic acid, F—formic acid. IV—iodine value (gI_2/100g), IV(%)—IV conversion, OOC—oxirane oxygen content (gO_2/100g), RCO(%)—relative conversion to oxirane, AV—acidity values (mgKOH/g).

Table S2. Physico-chemical parameters and conversion of fresh and wasted oils.

Entry	Oil	Indices						
		IV					AV	
		initial	after epoxi	IV(%)	OOC	RCO(%)	initial	after epoxi
1	OL	88 ± 1.8	27 ± 0.7	69.3 ± 0.3	3.4 ± 0.09	63.9 ± 0.15	3.4 ± 0.06	4.1 ± 0.06
2	MT	113.9 ± 2.3	25 ± 0.6	78.0 ± 0.4	4.9 ± 0.1	72.8 ± 1.2	9.0 ± 0.15	8.3 ± 0.3
3	SF	122 ± 3.0	50 ± 1.1	59.3 ± 0.1	3.9 ± 0.05	54.6 ± 0.5	2.1 ± 0.1	2.9 ± 0.1
4	HM	162.5 ± 4.0	41 ± 1.1	74.7 ± 0.3	6.5 ± 0.12	70.4 ± 0.6	3.7 ± 0.0	1.9 ± 0.1
5	LS	177 ± 1.9	43 ± 1.1	75.8 ± 0.5	7.1 ± 0.11	70.6 ± 1.1	3.6 ± 0.0	0.1 ± 0.0
6	Olw	84.3 ± 1.0	39 ± 1.0	54.4 ± 0.9	2.6 ± 0.03	52.1 ± 0.3	3.6 ± 0.06	1.0 ± 0.0
7	MTw	111.3 ± 2.2	54 ± 1.3	51.5 ± 0.4	2.8 ± 0.06	42.3 ± 0.3	9.7 ± 0.06	5.2 ± 0.06
8	SFw	115.7 ± 0.9	38 ± 0.9	67.1 ± 0.5	4.2 ± 0.06	62.1 ± 0.6	2.1 ± 0.0	1.2 ± 0.03
9	HMw	160.9 ± 2.2	64 ± 1.4	60.5 ± 0.3	4.8 ± 0.1	52.6 ± 0.1	4.2 ± 0.1	4.7 ± 0.12
10	LSw	176.8 ± 4.5	69 ± 1.8	61.1 ± 0.7	6.0 ± 0.1	60.3 ± 1.0	4.1 ± 0.1	1.5 ± 0.1

Experimental conditions: 2g oil, 1.5 NZ (wt%), molar ratio H_2O_2/double bonds/acid – 1:1:0.5; at 30°C and 6 h reaction time.

IV—iodine value (gI_2/100g), IV(%)—IV conversion, OOC—oxirane oxygen content (gO_2/100g), RCO(%)—relative conversion to oxirane, AV—acidity values (mgKOH/g).

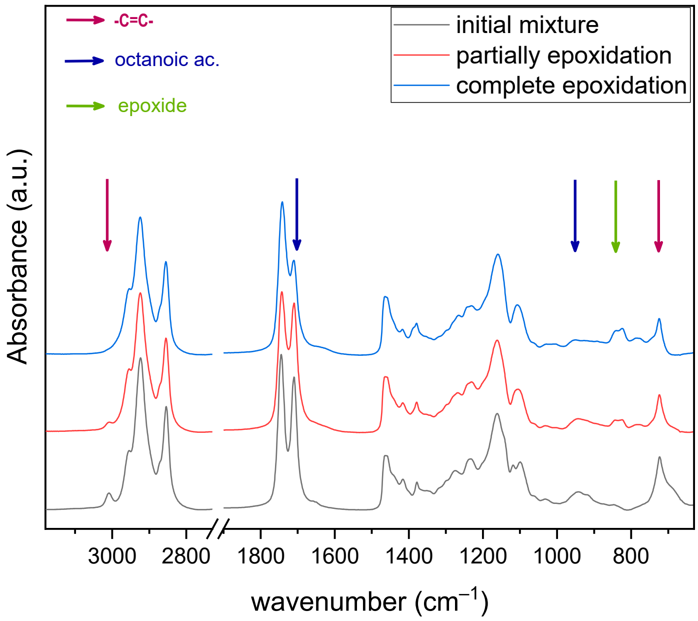

Figure S1. FTIR-ATR spectra for SFr before and after epoxidation: black line—initial mixture; red line—6 h, 30°C; blue line—12h, 30°C. Experimental conditions: 2g SFr, 1.5 wt% catalyst, molar ratio H_2O_2/double bonds/acid—1:1:0.5.

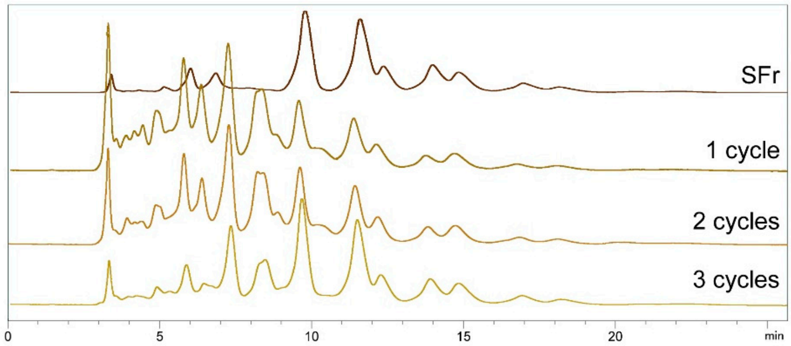

Figure S2. HPLC profile of the SFr after consecutive recycles of enzymatic epoxidation. Experimental conditions: 2g SFr, 1.5 wt% NZ, molar ratio H_2O_2/double bonds/peracid agent=1:1:0.5; at 30°C and 12 h reaction time.

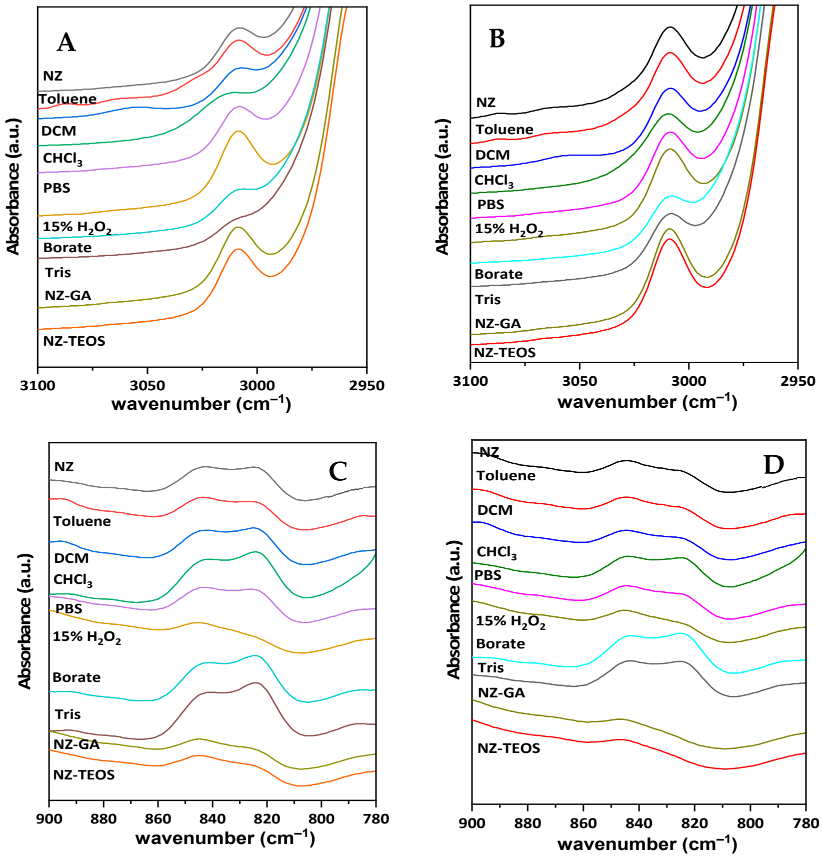

Figure S3. FTIR-ATR spectra of SFr samples: (A) -C=C—region after 1 cycle; (B) -C=C—region after 3 cycles; (C) epoxide region after 1 cycle; (D) epoxide region after 3 cycles. Experimental conditions: 2g SFr, 1.5 wt% biocatalyst, molar ratio H_2O_2/double bonds/octanoic acid=1:1:0.5. When an organic solvent or buffer is used, 0.5ml of the corresponding liquid was additionally added; at 30°C and 12 h reaction time.
